# Disorganized Innervation and Neuronal Loss in the Inner Ear of *Slitrk6*-Deficient Mice

**DOI:** 10.1371/journal.pone.0007786

**Published:** 2009-11-11

**Authors:** Kei-ichi Katayama, Azel Zine, Maya Ota, Yoshifumi Matsumoto, Takashi Inoue, Bernd Fritzsch, Jun Aruga

**Affiliations:** 1 Laboratory for Behavioral and Developmental Disorders, RIKEN Brain Science Institute (BSI), Wako-shi, Saitama, Japan; 2 University of Montpellier I, Institute of Neurosciences, INSERM U583, Montpellier, France; 3 Department of Biology, College of Liberal Arts and Sciences, University of Iowa, Iowa City, Iowa, United States of America; The University of Hong Kong, China

## Abstract

Slitrks are type I transmembrane proteins that share conserved leucine-rich repeat domains similar to those in the secreted axonal guidance molecule Slit. They also show similarities to Ntrk neurotrophin receptors in their carboxy-termini, sharing a conserved tyrosine residue. Among 6 *Slitrk* family genes in mammals, *Slitrk6* has a unique expression pattern, with strong expression in the sensory epithelia of the inner ear. We generated *Slitrk6*-knockout mice and investigated the development of their auditory and vestibular sensory organs. *Slitrk6*-deficient mice showed pronounced reduction in the cochlear innervation. In the vestibule, the innervation to the posterior crista was often lost, reduced, or sometimes misguided. These defects were accompanied by the loss of neurons in the spiral and vestibular ganglia. Cochlear sensory epithelia from *Slitrk6*-knockout mice have reduced ability in promoting neurite outgrowth of spiral ganglion neurons. Indeed the *Slitrk6*-deficient inner ear showed a mild but significant decrease in the expression of *Bdnf* and *Ntf3*, both of which are essential for the innervation and survival of sensory neurons. In addition, the expression of Ntrk receptors, including their phosphorylated forms was decreased in *Slitrk6*-knockout cochlea. These results suggest that Slitrk6 promotes innervation and survival of inner ear sensory neurons by regulating the expression of trophic and/or tropic factors including neurotrophins from sensory epithelia.

## Introduction

The Slitrk family consists of neuronal transmembrane proteins that control neurite outgrowth [1], [2]. Structurally, Slitrks share leucine-rich repeat (LRR) domains located amino-terminal to the transmembrane domain. LRR domains are present in many proteins and mediate protein–protein interactions [3]. LRR domains in the Slitrk family proteins are similar to those in all Slit family proteins, which control axon guidance and branching [4]. Tyrosine residues in the carboxy-terminus are another structural feature of the Slitrk family; these tyrosines are flanked by amino acid sequences similar to those in the carboxy-terminal domain of the Ntrk neurotrophin receptor [5].

The mouse *Slitrk* family contains 6 genes (*Slitrk1-6*) [1]. *Slitrk1* is required for higher brain functions [6]. However, little is known about the physiological roles of the other family members. Although *Slitrk1* through *Slitrk5* are expressed broadly throughout the brain, the expression of *Slitrk6* is highly restricted to thalamic nuclei [1]. Furthermore, a comprehensive expression analysis of *Slitrk6* revealed a strong expression in the auditory and vestibular sensory epithelia of the ear [7]. This unique expression pattern led us to investigate the role of *Slitrk6* in inner ear development. Inner ear sensory epithelia contain mechanosensory hair cells that recognize sound as well as linear and angular acceleration for balance [8]. During development, sensory epithelia also play an important role in the development of sensory neurons of the inner ear by releasing diffusible factors that promote survival and outgrowth of sensory neurons [9].

In the present study, we generated *Slitrk6*-knockout mice and investigated the development of their auditory and vestibular sensory organs. Histological examination and marker labeling studies revealed a reduction in the innervation density in the cochlea of *Slitrk6*-deficient mice. In addition, the innervation to the posterior crista was lost, reduced, or sometimes misguided in the vestibule. Many sensory neurons within the spiral and vestibular ganglia were lost during development. In the explant co-culture experiment, cochlear sensory epithelia from *Slitrk6*-knockout mice had less activity in promoting neurite outgrowth of the spiral ganglion neurons. These results indicate that Slitrk6 plays an important role in the sensory neural development of the inner ear by regulating the expression of molecules that promote survival and neurite outgrowth of sensory neurons.

## Results

### *Slitrk6* Expression during Inner Ear Development

**Figure 1:**
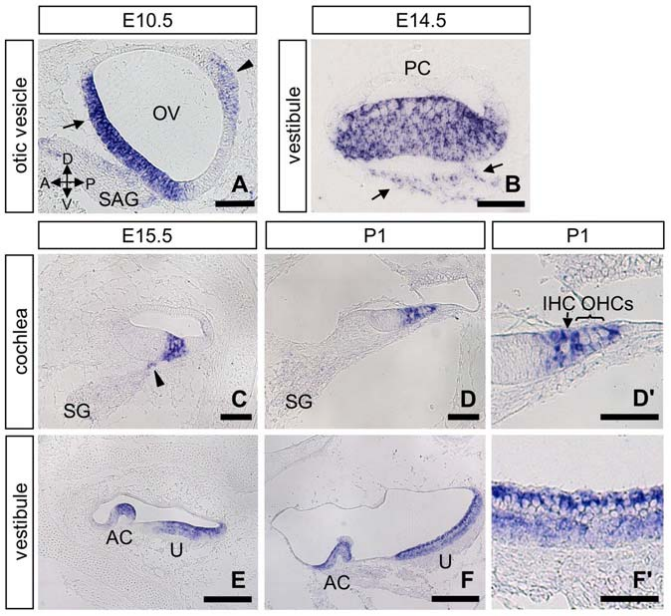
Expression of *Slitrk6* mRNA during inner ear development. In situ hybridization of *Slitrk6* at E10.5 (A), E14.5 (B), E15.5 (C, E), and P1 (D, D', F, F') in the otic vesicle (A), cochlea (C, D, D'), and vestibule (B, E, F, F'). *Slitrk6* transcripts are found in the ventromedial (arrow) and laterodorsal (arrowhead) regions of the otic vesicle at E10.5 (A). At E15.5, the expression of *Slitrk6* mRNA marks the region of the developing organ of Corti (C). In addition, a faint positive signal is seen in the nascent spiral ganglion neurons adjacent to the sensory epithelium (arrowhead in C). *Slitrk6* mRNA is still expressed in the organ of Corti at P1 (D), and higher magnification of the organ of Corti reveals that *Slitrk6* transcripts are localized densely in supporting cells and weakly in inner and outer hair cells (D'). *Slitrk6* mRNA is also detected in vestibular sensory epithelia, including ampullary cristae and utricular macula (B, E, F). Higher magnification of the utricular epithelium reveals that *Slitrk6* transcripts are densely located at the lumenal layer of the sensory epithelium (F'). A faint positive signal is also observed in the nascent vestibular ganglion neurons adjacent to the sensory epithelium (arrows in B). Anterior, posterior, dorsal and ventral directions are indicated by arrows in (A). AC, anterior crista; IHC, inner hair cell; OHC, outer hair cell; OV, otic vesicle; PC, posterior crista; SAG, statoacoustic ganglion; SG, spiral ganglion; U, utricle. Scale bars: A, B, C, D, D', F' 50 µm; E, F, 100 µm.

First, we carried out in situ hybridization analysis (Fig. 1) to know the *Slitrk6* transcripts distribution in the course of inner ear development. *Slitrk6* transcripts are first detected at embryonic day (E)8.5 in the otic placode, which invaginates to form the otic vesicle [7]. In the E10.5 otic vesicle, *Slitrk6* transcripts were strongly expressed in the ventromedial and laterodorsal regions (Fig. 1A), which give rise to the cochlear and vestibular sensory epithelia [8], [10], [11]. At E15.5, *Slitrk6* expression marked the presumptive organ of Corti (Fig. 1C). In addition, we detected relatively weak expression of *Slitrk6* in a narrow region of the spiral ganglion near the developing cochlear sensory epithelium from E12.5 to E15.5 (Fig. 1C). In the postnatal day (P)1 cochlea, *Slitrk6* transcripts were detected strongly in supporting cells and weakly in both inner and outer hair cells (Fig. 1D, D'). Furthermore, *Slitrk6*-positive signals were detected in vestibular sensory epithelia, including ampullary cristae, saccular and utricular maculae (Fig. 1B, E, F, and data not shown). *Slitrk6* transcripts were densely located at the lumenal surface of the sensory epithelium, where hair cells localize (Fig. 1F'). The hybridization signal was also detected weakly in supporting cells located at the basal cell layer of the vestibular sensory epithelium (Fig. 1F'). We also found weak expression of *Slitrk6* in vestibular ganglion neurons near the sensory epithelium from E11.5 to E14.5, comparable to what was observed in spiral ganglion neurons (Fig. 1B).

**Figure 2:**
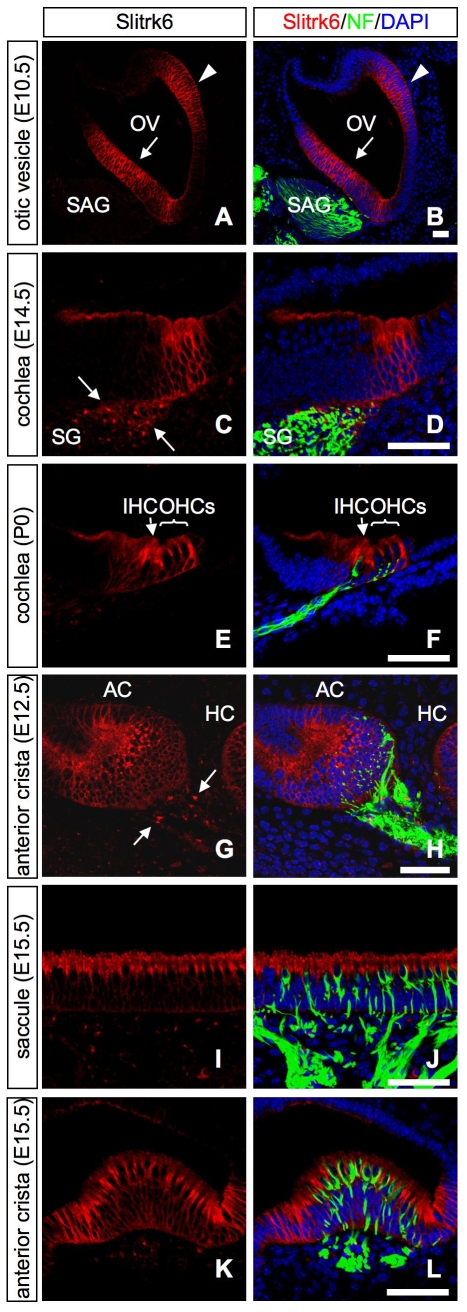
Expression of Slitrk6 protein during inner ear morphogenesis. Double immunohistochemistry for Slitrk6 (red; A–L), and neurofilament (NF, green; B, D, F, H, J, L) on transverse sections of the E10.5 otic vesicle (A, B), developing cochlea (C–F), and vestibular (G–L) sensory epithelia. Slitrk6 protein is found in the ventromedial (arrow) and laterodorsal (arrowhead) regions of the otic vesicle at E10.5 (A, B). At E14.5, Slitrk6 is strongly expressed in the area of the presumptive organ of Corti (C, D). The spiral ganglion neurons adjacent to the sensory epithelium are immunolabeled with Slitrk6 in a pattern that partly overlaps with neurofilament (arrows in C). At P0, Slitrk6 expression is confined to the supporting cells within the area of the organ of Corti (E, F). The neurofilament-labeled fibers project below the hair cells. In the macula of the sensory epithelium from an E15.5 saccule, Slitrk6 is uniformly and heavily distributed in the lumenal layer of the epithelium (I, J). In the sensory epithelium from E15.5 anterior crista, Slitrk6 expression is highly observed at the lumenal layer of the striolar region of the crista (K, L). At E12.5, faint Slitrk6-positive signals are also observed in the vestibular ganglion neurons adjacent to the sensory epithelium (arrows in G). DAPI-stained nuclei (blue) are seen in all panels of the merged images (B, D, F, H, J, L). AC, anterior crista; HC, horizontal crista; IHC, inner hair cell; OHC, outer hair cell; OV, otic vesicle; SAG, statoacoustic ganglion; SG, spiral ganglion. Scale bars, 50 µm.

Next, we examined the localization of the Slitrk6 protein using an anti-Slitrk6 antibody. Sltrk6–immunopositive signals were detected early in the otic vesicle (Fig. 2A, B), and later in cochlear and vestibular sensory epithelia where *Slitrk6* transcripts were distributed (Fig. 2C–L, Fig. S1, S2). In the presumptive organ of Corti, Slitrk6–immunopositive signals were strongly detected by E14.5 (Fig. 2C, D), and then confined to the supporting cell types at later stages of embryonic development and at the newborn (Fig. 2E, F, Fig. S2A–F). In the vestibular sensory epithelia, the signal was strong at the lumenal surface (Fig. 2G–L). However, double labeling with a hair cell marker calretinin indicated that the strong signals were in the non-hair cell region, presumably in the lumenal processes of the supporting cells (Fig. S2G–I). Transient expression of Slitrk6 in the spiral and vestibular ganglion neurons was also confirmed at the protein level, and positive signals were partly overlapped with neurofilament immunostaining (Fig. 2C, D, G, H). The cell type preferences of the *Slitrk6* expression in the cochlear and vestibular organs are comparable to each other.

### Generation of *Slitrk6*-Null Mutant Mice

**Figure 3:**
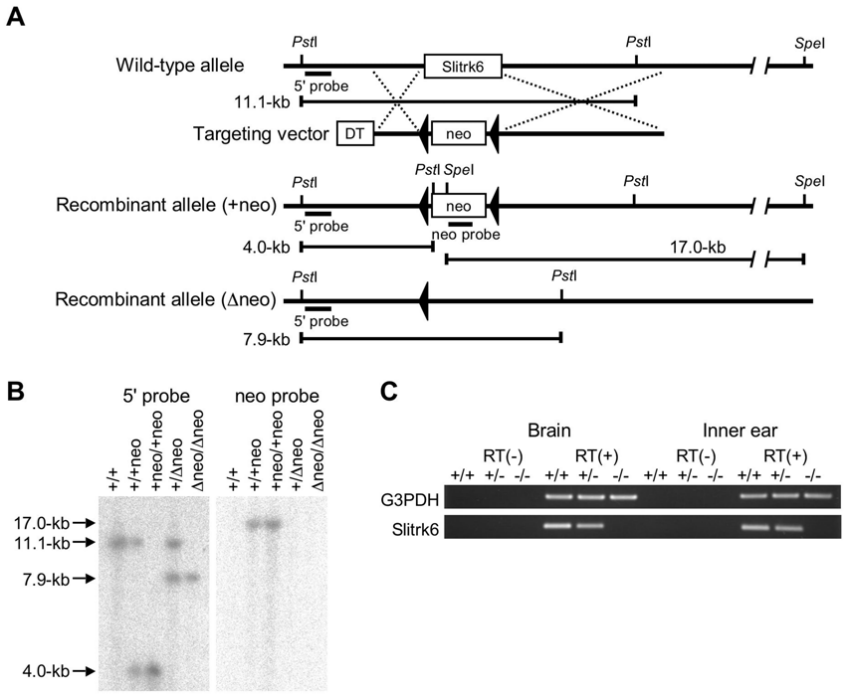
Targeted disruption of the *Slitrk6* gene. (A) Schematic structures of the *Slitrk6* genomic locus, targeting vector, and a mutated allele. The locations of the probes for Southern blotting (5′ and neo probes) are shown. DT, diphtheria toxin A; neo, neomycin-resistance gene. (B) Confirmation of homologous recombination of the mutant alleles by Southern blot. (C) RT-PCR performed on cDNAs prepared from brain stem and inner ear of *Slitrk6^+/+^*, *Slitrk6^+/−^*, and *Slitrk6^−/−^* mice at E16.5.

To analyze the role of Slitrk6 in inner-ear development, we generated *Slitrk6*-deficient mouse lines. A targeting vector was designed to replace the open reading frame (ORF) of *Slitrk6* with a phosphoglycerol kinase (PGK)-neo expression cassette flanked by a loxP sequence (Fig. 3A). We isolated 5 independent embryonic stem (ES) clones with homologous recombination, and 2 ES clones yielded chimeric mice capable of transmitting the disrupted allele (+neo) through the germline. Subsequently, the PGK-neo cassette was removed by crossing the heterozygous mice with mice transgenic for the *Cre recombinase* gene under the control of the cytomegalovirus immediate-early enhancer/chicken β-actin hybrid (CAG) promoter [12], which express *Cre recombinase* in their zygotes (Δneo, Fig. 3B). Ablation of *Slitrk6* mRNA was confirmed by RT-PCR (Fig. 3C). Heterozygous mating produced *Slitrk6* ^+/+^, *Slitrk6* ^+/−^, and *Slitrk6* ^−/−^ pups at the expected Mendelian frequencies. Both male and female mice grew without showing any external abnormalities and were fertile. The inner ear phenotypes described in the present study were observed in two mouse lines derived from two independent ES clones. Thus, the phenotypes are considered to correctly reflect the effects of the inserted mutation.

### Cochlear Innervation Defects in the Absence of *Slitrk6*

The gross morphology of the *Slitrk6*-deficient inner ear was normal. In addition, light microscopic analysis of hematoxylin and eosin (H&E)-stained sections showed that the general organization of the organ of Corti and the size of cochlear sensory epithelium in the *Slitrk6*-deficient mice was not obviously different from those of wild-type littermates (Fig. S3A, B, E, F).

**Figure 4:**
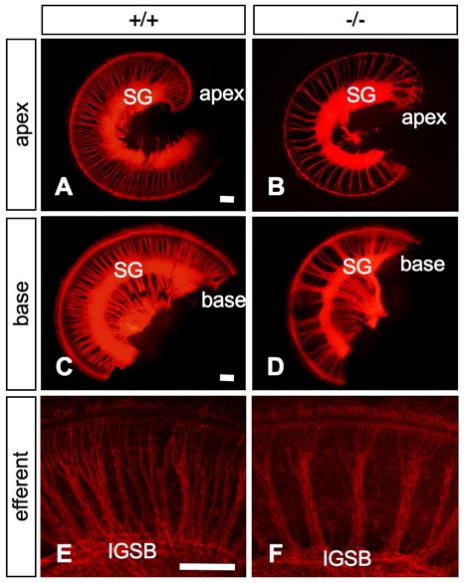
Cochlear innervation defects in *Slitrk6*-deficient mice. DiI-traced afferent innervation of apical (A, B) and basal (C, D) turns of the neonatal (P0) cochlea in *Slitrk6^+/+^* (A, C) and *Slitrk6^−/−^* (B, D) mice. *Slitrk6*-deficient inner ear displays a marked alteration in the spacing of radial fibers in the cochlear innervation (B, D). Innervation defects are distributed evenly throughout the cochlea with no basal-to-apical gradient. DiI-labeled efferent innervation of the cochlear middle turn of *Slitrk6^+/+^* (E) and *Slitrk6^−/−^* (F) neonatal mice. Efferent fibers show abnormalities similar to those noted for the afferent innervation. IGSB, intraganglionic spiral bundle; SG, spiral ganglion. Scale bars, 100 µm.

To initially characterize the innervation patterns of the inner ear in wild-type and mutant mice, we selectively labeled the afferent innervation of the cochleae at P0 by applying DiI to the alar plate of the brainstem, where the fibers terminate centrally. In contrast to the dense, evenly spaced innervation by radial fibers of the wild-type cochlea (Fig. 4A, C), the radial fibers in the *Slitrk6*-knockout mice were fasciculated with large spaces between the bundles (Fig. 4B, D). In *Slitrk6*-knockout mice, the innervation defects were distributed evenly throughout the cochlea, and all the cochlear turns were affected to the same degree (Fig. 4A–D).

To label the efferent fibers, we then placed DiI crystals in the olivo-cochlear efferent bundle near the floor plate [13], [14]. Selective DiI tracing of the olivo-cochlear efferents in the cochleae revealed that the efferent fibers were also abnormally fasciculated into fewer radial bundles, showing innervation defects similar to afferent fibers (Fig. 4E, F). Efferent fibers use afferent fibers as a scaffold during pathfinding, and the track of the efferents closely follows the afferent innervation pattern [15], [16]. Therefore, the observed cochlear efferent innervation defects are likely to be secondary to the afferent innervation defects.

**Figure 5:**
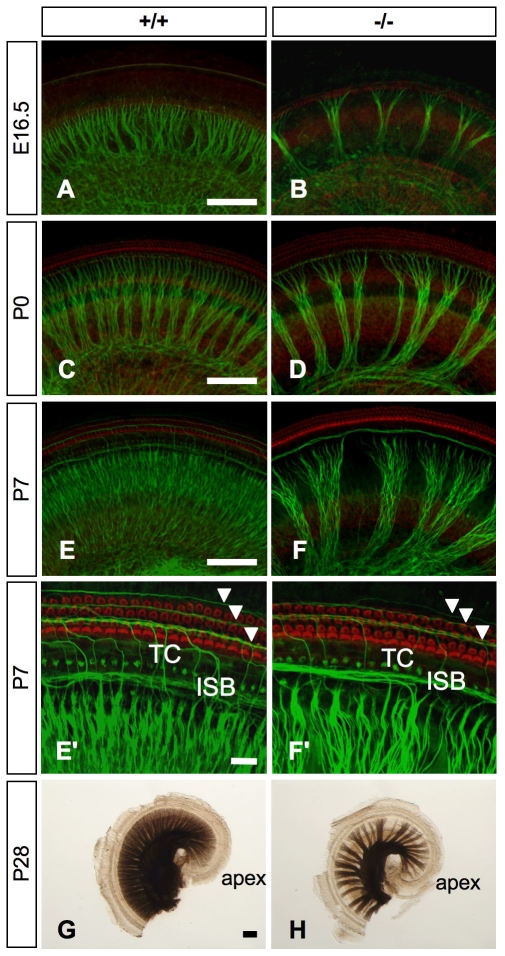
Patterns of cochlear innervation in whole-mount surface preparations of the cochlear sensory epithelia of wild-type and *Slitrk6*-deficient mice. Immunohistochemistry for neurofilament (green) in the mid-cochlear turn of *Slitrk6^+/+^* (A, C, E) and *Slitrk6^−/−^* (B, D, F) mice at E16.5 (A, B), P0 (C, D), and P7 (E, F). Specimens were stained with phalloidin-TRITC (red) to visualize the hair cells. Mutant mice show a marked reduction in the cochlear innervation as compared to wild-type mice. The density of radial fibers is reduced in the absence of *Slitrk6*. A higher magnification of the P7 cochlea indicates that the spiral fibers navigating in the outer hair cell area are also reduced in knockout mice (arrowheads in E', F'). Myelin-stained flat mounts of *Slitrk6^+/+^* (G) and *Slitrk6^−/−^* (H) cochleae at P28. The innervation defect of reduced radial fiber density is prominent in *Slitrk6*-deficient cochlea. ISB, inner spiral bundle; TC, tunnel of Corti. Scale bars: A, C, E, G, 100 µm; E', 20 µm.

We next examined the developmental changes of the cochlea innervation defects between E16.5 and P7, using whole-mount surface preparations of the cochlea labeled with anti-neurofilament antibody to visualize the innervation pattern (Fig. 5A–F). The radial fiber projection to the organ of Corti was strongly reduced in the mutant mice, leaving larger gaps between radial bundles than in the wild-type mice (Fig. 5A–F). At P7, a higher magnification view revealed that the type II spiral fibers that extend through the tunnel of Corti to the three rows of outer hair cells were reduced in number in *Slitrk6*-knockout mice (Fig. 5E', F'). Myelin staining by osmium tetroxide revealed that the defects in the innervation pattern were still prominent in *Slitrk6*-deficient mice at P28 (Fig. 5G, H).

### Vestibular Innervation Defects in the Absence of *Slitrk6*

We next examined the abnormalities in the vestibular region of *Slitrk6-*deficient mice. Light microscopic examination revealed that the sensory hair cells in all of the vestibular regions (ampullary cristae and saccular and utricular maculae) appeared normal with regard to their number, shape, and tissue architecture (Fig. S3C, D). However, the sensory epithelia in saccular macula and posterior crista were slightly smaller than those of wild-type littermates at E16.5, but not at E13.5 (Fig. S3E–G).

**Figure 6:**
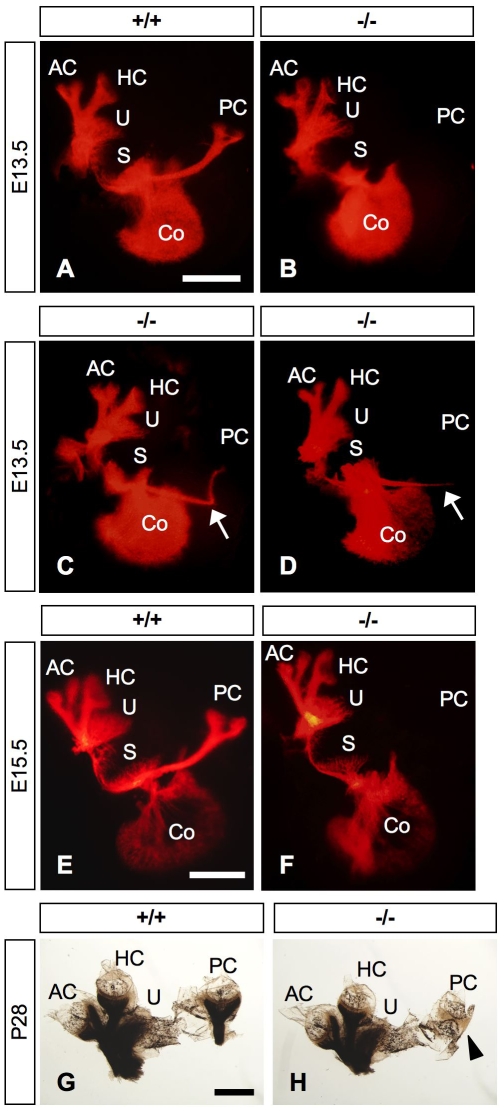
Vestibular innervation defects in *Slitrk6*-deficient mice. DiI-labeled flat mounts of *Slitrk6^+/+^* (A, E) and *Slitrk6^−/−^* (B–D, F) inner ears at E13.5 (A–D) and E15.5 (E, F). *Slitrk6*-deficient mice frequently lack innervation to the posterior crista (B, F), although, in some cases, the fiber projections to the posterior crista are misrouted (arrows in C, D). Myelin-stained flat mounts of *Slitrk6^+/+^* (G) and *Slitrk6^−/−^* (H) vestibule at P28; the whole innervation to the posterior crista is lacking (arrowhead). AC, anterior crista; Co, cochlea; HC, horizontal crista; PC, posterior crista; S, saccule; U, utricle. Scale bars, 500 µm.

DiI labeling of vestibular afferent fibers revealed that, from E13.5 to E15.5, innervation to the posterior crista was frequently absent in the *Slitrk6*-deficient inner ear (78.3% at E13.5 [n = 83] and 85.7% at E15.5 [n = 28]), and they sometimes showed reduced and abnormal trajectories (19.3% at E13.5 and 3.6% at E15.5) (Fig. 6A–F). At P28, myelin staining with osmium tetroxide confirmed that innervation to the posterior crista was still frequently absent in *Slitrk6*-knockout mice (Fig. 6G, H). After P14, 90.3% of posterior cristae of *Slitrk6*-deficient inner ear had no innervation (n = 62).

### Loss of Neurons in Spiral and Vestibular Ganglia of the *Slitrk6*-Deficient Mice

**Figure 7:**
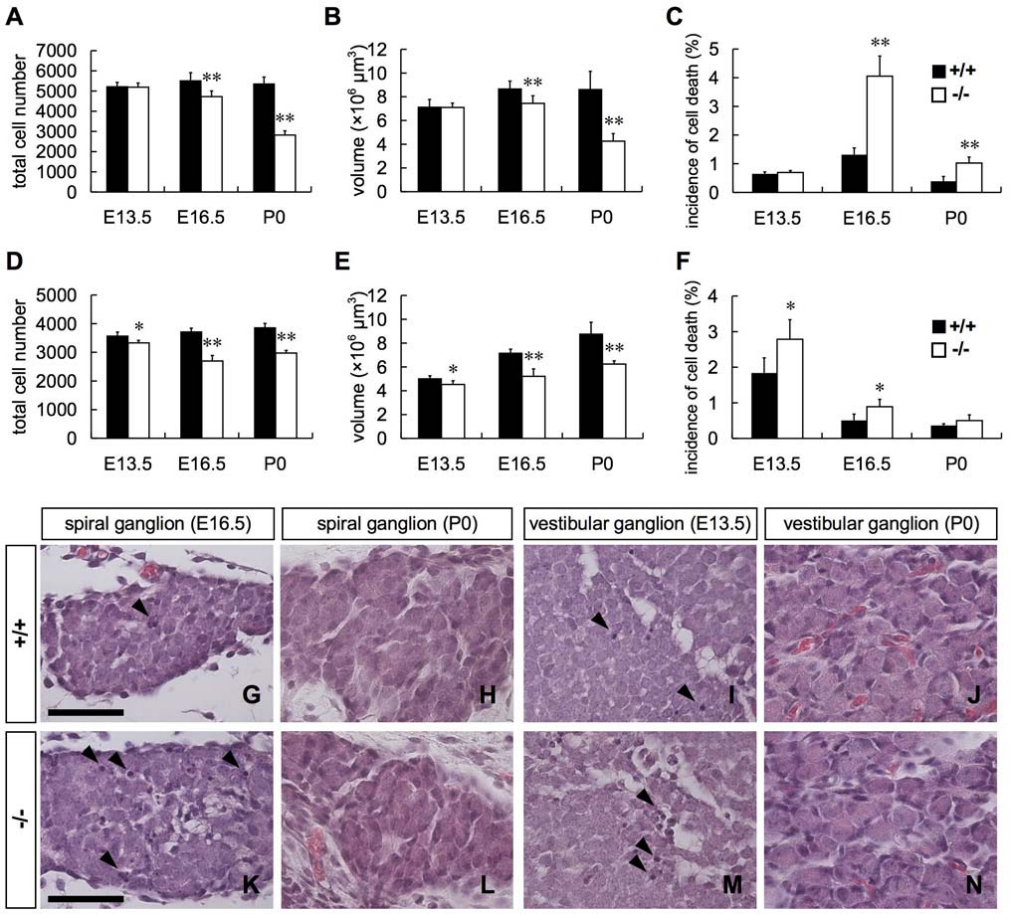
Neuronal loss in the spiral and vestibular ganglia in *Slitrk6*-deficient mice. Number of neurons (A, D), volume (B, E), and incidence of cell death (C, F) in the spiral (A–C) and vestibular (D–F) ganglia. Both the number of neurons and volume of the spiral ganglion of *Slitrk6*-knockout mice were significantly lower than those of wild-type mice at E16.5, and they decreased to about a half of those of wild-type mice at P0 (A, B). Cell death in the spiral ganglion significantly increased in *Slitrk6*-deficient mice at E16.5 and P0 (C). Both the number of neurons and the volume of the vestibular ganglion were significantly lower in the knockout than in the wild-type from E13.5, and both were about 75% of those of wild-type mice at E16.5 and P0 (D, E). Cell death in the vestibular ganglion was significantly increased in knockout mice at E13.5 and E16.5 (F). Measurements of *Slitrk6*-deficient vestibular ganglia were made in inner ears without posterior crista innervation. n = 5 to 7 in the spiral ganglion and n = 3 to 5 in the vestibular ganglion. Mean + SD, * *p*<0.05, ** *p*<0.01, Student's *t*-test. H&E-stained spiral ganglia at E16.5 (G, K) and P0 (H, L) and vestibular ganglia at E13.5 (I, M) and P0 (J, N) of *Slitrk6^+/+^* (G–J) and *Slitrk6^−/−^* mice (K–N). Pyknotic cells are frequently observed in the spiral ganglion of *Slitrk6*-deficient mice at E16.5 (arrowheads in G, K). In the vestibular ganglion, the number of pyknotic cells is significantly increased at E13.5 in mutant mice (arrowheads in I, M). At P0, there are no apparent differences between wild-type and knockout mice in cell size and organization in either the spiral or vestibular ganglia (H, J, L, N). Scale bars: 50 µm.

To examine whether the number of neurons in the spiral and vestibular ganglia is affected by the deletion of *Slitrk6*, we calculated the number of neurons in each ganglion and its volume. In the spiral ganglion, neither the number of neurons nor ganglion volume was affected at E13.5; however, both were significantly reduced at E16.5, and at P0, they decreased to about a half of those of wild-type mice (Fig. 7A, B). Cell death in the spiral ganglion was significantly higher in *Slitrk6*-deficient mice at E16.5 than in the wild-type (Fig. 7C, G, K, Fig. S4A, B). Although the incidence of cell death was markedly reduced at P0 relative to the incidence at E16.5, it was still significantly higher in *Slitrk6*-deficient than in wild-type mice. In contrast to the change in the spiral ganglion, in the vestibular ganglion both the number of neurons and the volume began to decrease from E13.5, and their values were about 75% of those of wild-type mice from E16.5 to P0 (Fig. 7D, E). The incidence of cell death in the vestibular ganglion was significantly higher in *Slitrk6*-knockout mice at E13.5 and E16.5 than in wild-type mice (Fig. 7F, I, M, Fig. S4C, D). At P0, H&E-stained sections showed no apparent differences between wild-type and knockout mice in cell size and organization in either the spiral or vestibular ganglion (Fig. 7H, J, L, N). Reduction in both the number of neurons and volume in vestibular ganglion was prominent in inferior vestibular ganglion that gives rise to posterior crista innervation (Fig. S5) [17], [18]. This result suggests that the neuronal loss within the vestibular ganglion is largely due to the loss of neurons that innervate the posterior crista.

### Decrease in the Neurite Outgrowth Activity in Cochlear Sensory Epithelia of *Slitrk6*-Deficient Mice

**Figure 8:**
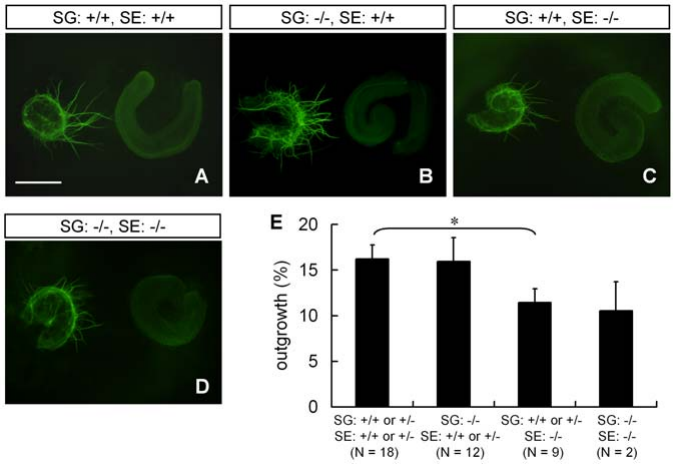
Cochlear sensory epithelia of *Slitrk6*-knockout mice have less activity in promoting neurite outgrowth of spiral ganglion neurons. Spiral ganglia (SG) and sensory epithelia (SE) were dissected from E14.5 wild-type and *Slitrk6*-deficient mice and the explants were cultured. Neurites were visualized by neurofilament immunostaining (green). There were hardly any detectable processes at the beginning of the explant culture. Spiral ganglia of wild-type (A) and *Slitrk6*-deficient (B) mice can strongly extend their neurites to sensory epithelia obtained from wild-type mice, whereas sensory epithelia of *Slitrk6*-knockout mice weakly attract neurites of spiral ganglia obtained from both wild-type (C) and knockout mice (D). Scale bar, 400 µm. (E) Measurement of neurite outgrowth (%) of spiral ganglia. The graphs represent mean + SEM. Neurite outgrowth of spiral ganglia was reduced in the presence of *Slitrk6*-deficient sensory epithelia compared to the presence of wild-type or heterozygous sensory epithelia. * *p*<0.05, Mann-Whitney's *U*-test.

Inner ear sensory epithelia are known to release diffusible factors that promote survival and outgrowth of statoacoustic ganglion neurons [9]. Thus we examined whether *Slitrk6*-deficient sensory epithelia promote the survival and outgrowth of ganglion neurons by using culture experiments. In the explant culture, spiral ganglion neurons of both wild-type and *Slitrk6*-knockout mice could extend neurites toward wild-type sensory epithelia (Fig. 8A, B, E). However, sensory epithelia of knockout mice weakly attracted the neurites of both wild-type and knockout spiral ganglia (Fig. 8C–E). These results suggest that there is a reduction in the expression of trophic and/or tropic factors in *Slitrk6*-deficient sensory epithelia.

### Decreased Expression of Neurotrophins and Their Receptors in the *Slitrk6*-Deficient Inner Ear

**Figure 9:**
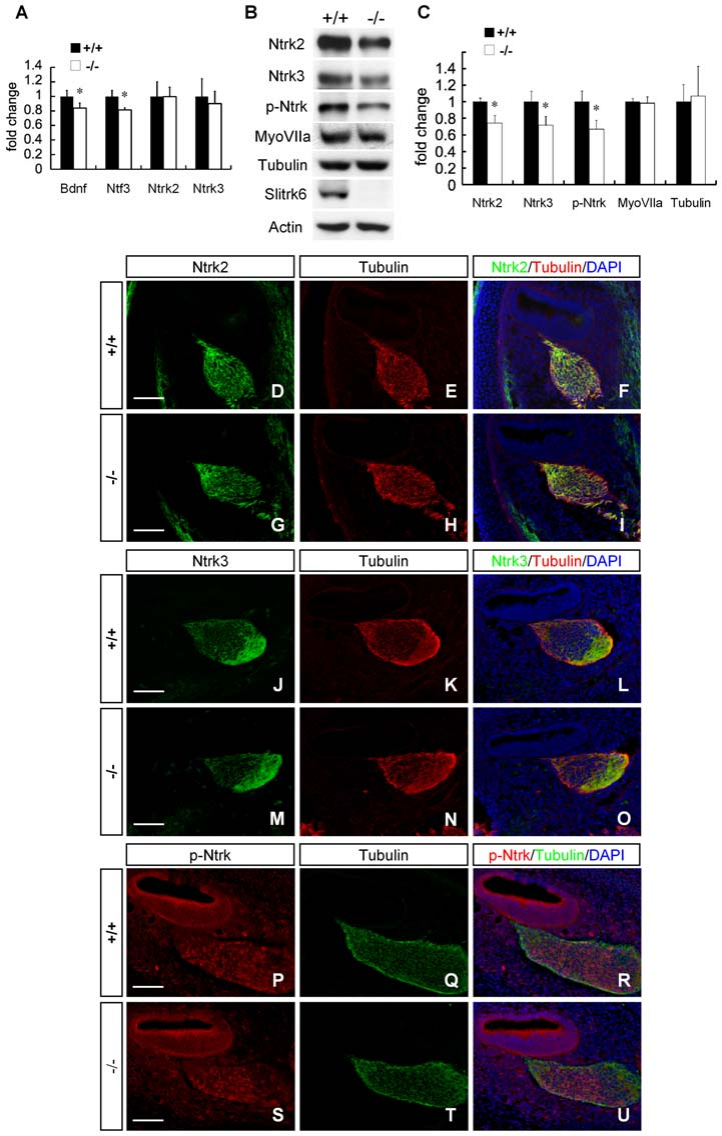
Expression of neurotrophins and their receptors was decreased in *Slitrk6*-deficient inner ear. (A) Real-time PCR analysis of *Bdnf*, *Ntf3*, *Ntrk2*, and *Ntrk3* in E14.5 inner ear (includes cochlear and vestibular sensory epithelia, and spiral and vestibular ganglia). The graphs depict mean + SD of 3 independent analyses. The mRNAs of *Bdnf* and *Ntf3* were significantly decreased in *Slitrk6*-deficient inner ear, whereas those of *Ntrk2* and *Ntrk3* were not. * *p*<0.05, Student's *t*-test. (B) Western blot of protein samples extracted from E14.5 cochlea (includes cochlear sensory epithelium and spiral ganglion). (C) Both Ntrk2 and Ntrk3 proteins in the cochlea were significantly decreased in *Slitrk6*-knockout mice. Furthermore, phosphorylated form of Ntrk (p-Ntrk) protein was decreased in the knockout mice. Amounts of a neuronal marker (βIII tubulin) and a sensory epithelium marker (Myosin VIIa) were comparable between the wild-type and knockout mice. The graphs represent mean + SD of 3 independent analyses. * *p*<0.05, Student's *t*-test. Immunohistochemistry for Ntrk2 (green; D, G), Ntrk3 (green; J, M), p-Ntrk (red; P, S), and βIII tubulin (red; E, H, K, N and green; Q, T) in mid-turn cochlea of *Slitrk6^+/+^* (D–F, J–L, P–R) and *Slitrk6^−/−^* (G–I, M–O, S–U) mice. Specimens were counterstained with DAPI (blue) and their merged images are shown in F, I, L, O, R, U. The spatial localizations of Ntrk2, Ntrk3 and p-Ntrk are not altered in knockout mice. Scale bars: 50 µm.

Among the molecules that promote survival and outgrowth of inner ear sensory neurons, neurotrophins are best characterized by multiple experiments. Brain-derived neurotrophic factor (Bdnf) and Neurotrophin-3 (Ntf3) are two neurotrophins that are produced in the developing sensory epithelia, and gene knockout studies revealed that they promote the innervation and support the survival of sensory neurons [19], [20]. Receptors for Bdnf (Ntrk2) and Ntf3 (Ntrk3) are expressed in sensory neurons, and mice deficient in these receptors also show reduced innervation and the loss of sensory neurons that mirror the ligand defects [21]. Therefore, we analyzed the expression of these molecules in E14.5 inner ear (which included the cochlear and vestibular sensory epithelia and the spiral and vestibular ganglia) by using real-time PCR. The expression of *Bdnf* and *Ntf3* mRNA was significantly decreased in *Slitrk6*-deficient inner ear, whereas the expression of *Ntrk2* and *Ntrk3* was not affected (Fig. 9A). In situ hybridization of *Bdnf* and *Ntf3* did not show any differences in the distribution of these mRNAs between wild-type (data not shown). We further examined the expression of Ntrk receptors using protein samples extracted from E14.5 cochleae (which included the cochlear sensory epithelium and spiral ganglion). We used cochleae at this developmental stage because neuronal loss in the spiral ganglion might affect the results at later stages. The expression level of both Ntrk2 and Ntrk3 proteins was significantly decreased in *Slitrk6*-deficient cochleae (Fig. 9B, C). In addition, the amount of phosphorylated form of Ntrk receptor was decreased in knockout mice (Fig. 9B, C). However, their spatial localization was not altered in the knockout mice (Fig. 9D–U). This suggests that Slitrk6 deletion interferes with the level of neurotrophin mRNA but affects Ntrks predominantly at the post-transcriptional level.

## Discussion

In the present study, we developed *Slitrk6*-knockout mice and analyzed the development of their auditory and vestibular sensory organs. The overall innervation density of the cochlea was markedly reduced in the *Slitrk6*-deficient mice, and the innervation to the posterior crista in the vestibule was reduced, disoriented, or lost. Many sensory neurons in both the spiral and vestibular ganglia died during late embryonic development.

Slitrk6 is predominantly expressed in the sensory epithelia, which release trophic and/or tropic factors for sensory neurons. Especially, supporting cells of the organ of Corti in which Slitrk6 is strongly expressed, have been shown to play an important role in the survival of cochlear sensory neurons [22], [23]. The present results suggest that Slitrk6 participates in the transmembrane signal transduction processes that enhance the expression of such factors in sensory epithelia. Among these factors, neurotrophins are best understood by numerous studies [19], [20], [21]. In the developing inner ear, Ntf3 is expressed predominantly in the supporting cell and Bdnf in the hair cell lineages, and both Ntrk2 and Ntrk3 are expressed in the sensory neurons [22], [24], [25]. Mice deficient in one of these neurotrophins or Ntrk receptors show reduced innervation and loss of sensory neurons (Fig. S6) [16], [26]–[29]. *Slitrk6*-deficient inner ear showed mild but significant decrease in the expression level of both *Bdnf* and *Ntf3* (Fig. 9A). In addition, we found the decrease in the expression of both Ntrk2 and Ntrk3 at protein level, and the amount of phosphorylated form of Ntrk receptors also decreased in *Slitrk6*-deficient cochlea (Fig. 9B, C). These results suggest a disturbance in the neurotrophin-Ntrk signaling in *Slitrk6*-deficient inner ear. Indeed, there are some similarities in the inner ear phenotypes of *Slitrk6*- and neurotrophin/Ntrk-deficient mice (Fig. S6, Table S1). Although the phenotypes of the inner ear with a partial reduction of both Bdnf-Ntrk2 and Ntf3-Ntrk3 signalings are still unknown, *Bdnf* heterozygous mutant mice display an intermediate decrease in size and neuronal number of the vestibular ganglion [28]. Thus, it is likely that quantitative changes in neurotrophin signaling contribute to the development of the inner ear phenotypes of *Slitrk6*-knockout mice.

Together with the co-culture experiment (Fig. 8), the reduction of the neurotrophins from cochlear sensory epithelia may be primarily responsible for the spiral ganglion innervation defects. This idea is also supported by a result that isolated spiral ganglia of *Slitrk6*-knockout mice can extend their neurites almost the same as those of wild-type mice in the presence of Ntf3 (Fig. S7). On the other hand, the sizes of the sensory epithelia in saccular macula and posterior crista were slightly lower in *Slitrk6*-deficient mice compared to those of wild-type littermates in late embryonic stages (Fig. S3E, F). This result indicates that Slitrk6 play some unidentified roles in the development of sensory epithelia per se. However, we believe that the innervation defects are not due to the sensory epithelial size reduction, because the innervation defect to the posterior crista was observed as early as E13.5 when the size reduction in the epithelium was not apparent.

The results in the present study indicate the involvement of neurotrophin–Ntrk signaling in the inner ear defects of *Slitrk6*-knockout mice. However, the decrease in the expression of neurotrophins in *Slitrk6*-knockout mice was relatively small compared to their dramatic phenotype. Culture experiments identified several candidate molecules (TGF-β, LIF, MCP-1, erythropoietin and Fgf2) that affect the survival and outgrowth of inner ear sensory neurons [30]–[33]. Slitrk6 may regulate the expression of some of those or other unidentified trophic and/or tropic factors that promote neuronal survival and neurite extension in addition to neurotrophins. Decrease in the expression of such factors may also affect the dynamics of Ntrk neurotrophin receptors.

Several genes are known to be critical for sensory neural development in the mouse inner ear, including *NeuroD1*, *Brn3a* (also called as *Pou4f1*), and *ErbB* [34]–[38]. Mice lacking *NeuroD1* or *Brn3a* display defects in posterior crista innervation similar to those in *Slitrk6*-knockout mice, as well as loss of the inner ear sensory neurons [34]–[36]. The expression of Ntrk neurotrophin receptors is decreased in *NeuroD1*- and *Brn3a*-knockout mice, suggesting that neurotrophin-Ntrk receptor signaling is involved in the innervation defects and neuronal loss in these mutant mice [34]–[36]. Mice lacking ErbB function also show a marked decrease in the number of spiral ganglion neurons and disorganized inner ear innervation [37], [38]. ErbB2 and ErbB3 are highly expressed in the supporting cells of the organ of Corti and regulate the expression of neurotrophins, especially *Ntf3*. However, we could not detect any significant alteration in the expression of NeuroD1, Brn3a, ErbB2, or ErbB3 in the E14.5 cochleae (Fig. S8), in contrast to the reduction of neurotrophin receptors (Fig. 9B, C). Therefore, these genes may not function downstream of Slitrk6 during inner ear development.

Besides the strong expression in the sensory epithelia, Slitrk6 is also expressed in ganglion neurons weakly and transiently. However, isolated spiral ganglia of *Slitrk6*-knockout mice can extend their neurites almost the same as those of wild-type mice in a tissue culture experiment (Fig. 8A, B, E, Fig. S7). Although we cannot exclude the possibility that Slitrk6 expressed in ganglion neurons play some roles in target recognition or axon pathfinding, we consider that the observed abnormalities of *Slitrk6*-knockout mice are mainly attributed to the loss of Slitrk6 in sensory epithelia. Interestingly, previous studies have also reported the transient expression of neurotrophins in the inner ear ganglia, similar to the expression of Slitrk6 [22], [25]. The neurons that transiently express neurotrophins are cells delaminating from the sensory epithelia [39], and are postulated to have a role in guiding neurites from sensory neurons back to the area they delaminated from [40]. On the basis of its spatial distribution, we believe that Slitrk6 is localized in the delaminating cells, where it could participate in the neuronal organization of the inner ear and provides guidance as shown in the derailed pathfinding of posterior crista projections.

In conclusion, our results indicate that Slitrk6 is involved in the survival and innervation of sensory neurons in the inner ear, at least in part by modulating neurotrophin–Ntrk signaling. The molecular mechanisms involved in auditory and vestibular axon pathfinding are beginning to be unraveled [41], [42]. The results presented here provide new insight into the mechanisms of innervation in the developing inner ear. Further investigation of the molecular mechanisms of Slitrk6 function will increase our knowledge of sensory neural development of the inner ear.

## Materials and Methods

### Animals

Animal experiments were approved by the Animal Experiment Committee of the RIKEN Brain Science Institute. The mice were maintained by the Laboratory Animal Facility, Research Resource Center, RIKEN Brain Science Institute.

### Generation of *Slitrk6*-Null Mutant Mice

*Slitrk6*-null mutant mice were generated as described previously [6], [43]. Briefly, to construct the *Slitrk6* targeting vector, overlapping *Slitrk6* genomic clones were isolated from a phage library made from mice of the 129SV strain (Stratagene, La Jolla, CA). The targeting construct contained the 1.8-kb 5′ and 5.3-kb 3′ homology regions, and the 3.3-kb fragment containing the ORF of *Slitrk6* was replaced with a floxed PGK-neo expression cassette (Fig. 3A). E14 ES cells were transformed with the targeting construct by electroporation and selected with G418. Drug-resistant clones were screened by Southern blot analysis using *Pst*I- and *Spe*I-digested genomic DNA hybridized, respectively, with a 0.7-kb 5′ genomic fragment corresponding to a genomic sequence outside of the sequence used in the targeting vector and a 0.6-kb *Pst*I PGK-neo probe (Fig. 3A). Chimeric mice were generated by the injection of targeted ES cells into C57BL/6J blastocysts. To excise the PGK-neo cassette, mice with germline transmission of the transgene were first mated with CAG-Cre mouse. The correct excision of the PGK-neo cassette was confirmed by Southern blot analysis (Fig. 3B). The mice carrying the mutated *Slitrk6* allele were backcrossed to C57BL/6J for more than 4 generations before analysis. Genotyping of progeny was performed by Southern blot or PCR analysis of genomic DNA extracted from tails. The PCR primers used were Slitrk6S (5′-CAGGAAGCATTCTCTTCTCTGTGA-3′), Slitrk6WTAS (5′-CATTGGTGACTGGGACTGTAGA-3′), and Slitrk6KOAS (5′-GGAAGCTCAGAACACTTACCTG-3′).

### Generation of an Anti-*Slitrk6* Antibody

A polyclonal anti-Slitrk6 antibody was raised in a rabbit against peptides corresponding to the carboxy-terminal region of mouse Slitrk6 (KNEYFELKANLHAEPDYLEVLEQQT). Peptides were synthesized and conjugated to keyhole limpet hemocyanin through a cysteine added to the N-terminus of the peptide. After immunization by conventional methods, antiserum was obtained and the antibody was purified by affinity chromatography with the immunized peptide. The antibody specificity was confirmed by the absence of Slitrk6 immunopositive signals in the *Slitrk6*-deficient inner ear (Fig. S1, Fig. 9B).

### Morphological Analyses

In situ hybridization analysis for *Slitrk6* was performed on paraformaldehyde-fixed paraffin sections obtained from wild-type mice as described previously [1], [7].

Immunohistochemistry was performed on paraffin or frozen sections and whole mounts. Rabbit polyclonal anti-Slitrk6 and anti–phospho-Ntrk (phosphorylated Tyr490 in NTRK1 and its corresponding residues in Ntrk2 and Ntrk3, Cell Signaling Technology, Beverly, MA), goat polyclonal anti-Ntrk2 (R&D Systems, Minneapolis, MN) and anti-Ntrk3 (R&D Systems), and mouse monoclonal anti-neurofilament (NF, Sigma, St. Louis, MO) and anti-βIII tubulin (Promega, Madison, WI) antibodies were used as primary antibodies. Alexa488 or 594-conjugated anti-rabbit, -goat, and -mouse IgG (Molecular Probes, Eugene, OR) were used as the secondary antibodies. To visualize inner and outer hair cells, whole mounts were stained with phalloidin-tetramethylrhodamine B isothiocyanate (TRITC) (Sigma). For the visualization of myelinated nerve fibers, relatively mature inner ears were impregnated with osmium tetroxide.

To reveal the general pattern of innervation, nerve fibers were visualized with a lipophilic tracer. DiI (1,1′-dioctadecyl-3,3,3′,3′-tetramethylindocarbocyanine perchlorate; Molecular Probes) crystals were placed into the brainstem alar plate to label eighth nerve afferent fibers to the inner ear, or into the olivo-cochlear efferent bundle near the floor plate to label the efferent fibers [13], [14]. After an appropriate diffusion time of 3 to 5 days at 37°C, whole inner ears or cochleae were then dissected out and viewed as whole-mount surface preparations. All the specimens mentioned above were examined with an Axioscop 2 plus (Carl Zeiss, Göttingen, Germany), MZ 16 FA (Leica Microsystems, Wetzlar, Germany), or FV1000 confocal laser microscope (Olympus, Tokyo, Japan).

For morphometrical analyses, inner ears from wild-type and *Slitrk6*-deficient mice were fixed in paraformaldehyde, embedded in paraffin, and serially sectioned at 6 µm. Every fourth section was stained with H&E. Comparisons of neuronal numbers in the spiral and vestibular ganglia were made by counting the number of neurons with clear nucleoli. No correction was made for split nucleoli. Dying neuronal cells that could be identified by pyknotic nuclei were counted in the same way. The volume of each ganglion was calculated by measuring the area occupied by neurons in each section using AxioVision software (Carl Zeiss).

### Explant Culture of Cochlear Sensory Epithelium and Spiral Ganglion

Sensory epithelium and spiral ganglion were dissected from E14.5 cochlea. A cochlear sensory epithelium and a spiral ganglion were randomly chosen and placed in three-dimensional collagen gel (Cell Matrix; Nitta Gelatin, Osaka, Japan) about 500 µm apart each other. Then they were cultured in neurobasal medium (Invitrogen, Carlsbad, CA) containing 2 mM L-glutamine, B27 supplement (Invitrogen) for 48 hours. Neurites were visualized by the immunostaining for neurofilament (Sigma). Neurite outgrowth was analyzed with ImageJ (http://rsb.info.nih.gov/ij/). Area occupied by the neurofilament-positive neurites were measured and relative to the area of the ganglion body (%) is presented.

### RNA Extraction and Real-Time RT-PCR Analyses

Inner ears from 5 to 6 E14.5 wild-type and *Slitrk6*-deficient mice were pooled, and total RNA was extracted with an RNeasy Protect Kit (QIAGEN, Hilden, Germany). Reverse transcription was performed with Superscript II reverse transcriptase (Invitrogen). Real-time PCR was performed with SYBR Green Real-time PCR Master Mix (Applied Biosystems, Foster City, CA) on an ABI Prism 7900HT (Applied Biosystems). The primers used for detection of *Bdnf* and *Ntf3* were designed by Stankovic and Corfas [44], and those for *Ntrk2* and *Ntrk3* were as follows: *Ntrk2*, sense: 5′-AGCCTTCTCCAGGCATCGT-3′, antisense: 5′-TCCGGGTCAACGCTGTTAG-3′, and *Ntrk3*, sense: 5′-TGTAGTTTCTGGCGGATTTTCTT-3′, antisense: 5′-AGCACGGAGCCCACATAGTC-3′. The intensity relative to *G3PDH* was calculated, and the fold change relative to the relative intensity in wild-type mice is presented.

### Protein Extraction and Western Blot Analysis

Cochleae from 12 to 13 E14.5 wild-type and *Slitrk6*-deficient mice were dissected and homogenized in RIPA buffer (50 mM Tris-HCl pH 7.8, 150 mM NaCl, 1% NP-40, 0.5% sodium deoxycholate, 0.1% SDS, 1 mM EDTA, 2 mM NaF, 1 mM Na_3_VO_4_ and protease inhibitor cocktail [Roche Diagnostics, Mannheim, Germany]). Approximately 20 µg of extract was loaded onto a SDS-PAGE gel, electrophoresed, and transferred to a polyvinylidene fluoride membrane (Millipore, Billerica, MA). Rabbit polyclonal anti-Slitrk6, anti–phospho-Ntrk (Cell Signaling Technology), anti–Myosin VIIa (Abcam, Cambridge, UK), anti–β-actin (Sigma), goat polyclonal anti-Ntrk2 (R&D Systems), and anti-Ntrk3 (R&D Systems), and mouse monoclonal anti-βIII tubulin (Promega) antibodies were used as primary antibodies. After incubation with appropriate secondary antibodies conjugated to horseradish peroxidase, the signals were detected with an ECL Plus kit (GE Healthcare, Buckinghamshire, UK). Signals in the Western blots were analyzed with ImageJ. The relative intensity against β-actin was calculated, and fold change relative to the relative intensity in wild-type mice is presented.

## Supporting Information

Table S1 Comparison of the phenotypes of *Slitrk6*-knockout mice and those of neurotrophin/Ntrk knockout mice.
(0.03 MB DOC)

Figure S1 Immunohistochemistry for *Slitrk6*. The polyclonal antibody raised against the carboxy-terminal region of mouse *Slitrk6* specifically recognizes endogenous *Slitrk6* protein. Cochlea (A, D), saccule (B, E), and anterior crista (C, F) of *Slitrk6* ^+/+^(A–C) and *Slitrk6* ^−/−^ (D–F) mice at P0. Positive signals on sensory epithelia disappear in *Slitrk6*-deficient mice. Scale bar, 50 µm.
(0.69 MB PDF)

Figure S2 Immunostaining of newborn inner ear sensory epithelia with *Slitrk6* and epithelium cell type markers. Double immunohistochemistry for *Slitrk6* (green; A, D, G), and supporting cell marker p27 (red; C) or hair cell markers Calbindin (red; F) and Calretinin (red; I) on transverse sections of the P0 organ of Corti (A–C), P1 organ of Corti (D–F) and saccular macula (G–I). Immuno-staining was carried out on paraffin sections by using rabbit polyclonal anti-*Slitrk6*, and mouse monoclonal anti-p27 (BD Transduction Labs, San Diego, CA), anti-Calbindin (Sigma) and anti-Calretinin (Millipore) antibodies. Merged images are shown in B, E, H and DAPI-stained nuclei are seen in E, H. Arrowheads in G–I indicate the strong *Slitrk6* signals outside the hair cell marker signals. Scale bar, 50 µm.
(0.69 MB PDF)

Figure S3 Histological examination of the inner ear sensory epithelia. H&E-stained sections of organ of Corti (A, B), and anterior crista and utricle (C, D) of *Slitrk6* ^+/+^ (A, C) and *Slitrk6* ^−/−^ (B, D) mice at P0. Higher magnifications of A, B, C, and D are shown in A', B', C', C', D' and D'. The organization of the organ of Corti in the *Slitrk6*-deficient mice is not clearly different from that of wild-type littermates in a light microscopic analysis of H&E-stained sections (A, B). In both the wild-type and *Slitrk6*-deficient mice, one row of inner hair cells and three rows of outer hair cells can be identified in all turns, and the differentiation of these cells in the mutant animals appears normal (A', B'). The organization of the anterior crista and utricle also appears normal in *Slitrk6*-deficient mice (C, D). Higher magnification views of the anterior crista (C', D') and utricle (C', D') are not clearly different between wild-type and knockout mice. Scale bars, 50 µm. Sizes of sensory epithelia in wild-type (+/+) and *Slitrk6*-deficient (−/−) at E13.5 (E; +/+, N = 5; −/−, N = 5) and E16.5 (F; +/+, N = 7; −/−, N = 7). The sizes were measured in every fourth of serially prepared H&E-stained sections (thickness: 6 µm) that include the entire sensory epithelia. The sizes are indicated as the summed lumenal surface lengths of the sensory epithelia in the serial sections. A common arbitrary unit is used between (E) and (F). * *p*<0.05, ** *p*<0.01, Student's *t*-test. (G) Densities of the hair cells in the vestibular sensory epithelia at E16.5. The cell densities were measured by counting Myosin VIIa immuno-stained cells per unitary length (1 mm) in the serial sections. There were no significant differences in the hair cell densities between wild-type and *Slitrk6*-deficient mice. AC, anterior crista; Co, cochlea; HC, horizontal crista; IHC, inner hair cell; OHC, outer hair cell; PC, posterior crista; S, saccular macula; SG, spiral ganglion; U, utricle.
(0.69 MB PDF)

Figure S4 Confirmation of the cell death in the spiral and vestibular ganglia by the TUNEL method. TUNEL-stained sections of the cochlea (A, B) at E16.5, and vestibular ganglia at E13.5 (C, D) of *Slitrk6* ^+/+^ (A, C) and *Slitrk6* ^−/−^ (B, D) mice. TUNEL staining was carried out using Apoptag peroxidase in situ apoptosis detection kit (Chemicon, Temecula, CA) in accordance with the manufacturer's instructions. The positive signals were visualized by a peroxidase-diaminobenzidine reaction, and then the sections were counterstained with methyl green. TUNEL-positive signals are increased in *Slitrk6*-deficient mice in both the spiral and vestibular ganglia. SG, spiral ganglion. Scale bars, 50 µm.
(0.69 MB PDF)

Figure S5 Reduction in both the number of neurons and volume of vestibular ganglion in *Slitrk6*-deficient mice was more pronounced in inferior vestibular ganglion than superior vestibular ganglion. Number of neurons (A) and volume (B) of the vestibular ganglion at P0. Values are presented as Mean + SD of 4 wild-type (black bar) and 3 knockout (white bar) mice. *, *p*<0.05; **, *p*<0.01 in Student's *t*-test.
(0.69 MB PDF)

Figure S6 Comparisons of the inner ear phenotypes of *Slitrk6*-knockout mice and those of neurotrophin/Ntrk-knockout mice. Schematic drawing of the inner ear phenotypes of wild-type (A), *Slitrk6*-knockout (B), *Bdnf/Ntrk2*-knockout (C) and *Ntf3/Ntrk3*-knockout mice (D). Cristae and maculae are indicated in yellow. Vestibular ganglia and their projections are shown in orange and spiral ganglia are shown in red. The number of vestibular ganglion neurons is severely decreased in *Bdnf*- and *Ntrk2*-null mutants (C), whereas a reduction in the number of the spiral ganglion neurons (indicated by thick red lines in cochlea) is prominent in *Ntf3*- and *Ntrk3*-null mutants (D) [21], [26], [27]. In the cochlea, *Ntf3*- and *Ntrk3*-null mutants predominantly lose spiral ganglion neurons in the basal turn (D), and neuronal loss in *Bdnf*- and *Ntrk2*-null mutants is most obvious in the apex (C) [16], [21], [28], [29]. In the *Slitrk6*-knockout mice, neuronal loss was more pronounced in the cochlea than in the vestibule, but *Slitrk6*-deficient cochleae showed no phenotypic gradient in neuronal loss along the baso-apical axis (B). AC, anterior crista; HC, horizontal crista; PC, posterior crista; S, saccule; SG, spiral ganglion; U, utricle; VG, vestibular ganglion.
(0.69 MB PDF)

Figure S7 Spiral ganglion neurons of *Slitrk6*-deficient mice can extend neurites identical to those of wild-type mice in the presence of neurotrophin. Spiral ganglia from E14.5 wild-type (+/+) and *Slitrk6*-knockout (−/−) mice were embedded in the collagen gel (Nitta Gelatin) and cultured in neurobasal medium (Invitrogen) containing 2 mM L-glutamine, B27 supplement (Invitrogen) and 20 ng/ml of Neurotrophin-3 (+Ntf3, PeproTech EC, London, UK) for 48 hours. Neurites were visualized by neurofilament immunostaining (Sigma, green). Spiral ganglion neurons of *Slitrk6*-knockout mice (B) can extend their neurites as those of wild-type mice (A). Scale bar, 200 µm. (C) Measurement of neurite outgrowth (%) of spiral ganglia. The graphs represent mean + SEM. Neurite outgrowth of spiral ganglia was not significantly different between wild-type and *Slitrk6*-deficient mice.
(0.69 MB PDF)

Figure S8 Amounts of proteins that are known to mediate inner ear sensory neural development in *Slitrk6*-knockout mice. (A) Western blot analysis of proteins extracted from E14.5 cochlea (includes cochlear sensory epithelium and spiral ganglion). Goat polyclonal anti-NeuroD1 (Santa Cruz Biotechnology), mouse monoclonal anti-Brn3a (Santa Cruz Biotechnology) and anti-ErbB3 (Thermo Fisher Scientific, Fremont, CA), and rabbit polyclonal anti-ErbB2 (Thermo Fisher Scientific) and anti-β-actin (Sigma) antibodies were used as primary antibodies. (B) The graphs represent the mean + SD of 3 independent analyses. There were no significant differences in the expression of NeuroD1, Brn3a, ErbB2, or ErbB3 between wild-type (+/+) and *Slitrk6* knockout (−/−) mice.
(0.69 MB PDF)
