# Supplementary material for: Disorganized Innervation and Neuronal Loss in the Inner Ear of Slitrk6-Deficient Mice
Source: PLoS One. 2009 Nov 11;4(11):e7786. doi: 10.1371/journal.pone.0007786 (PMC2777407; doi:10.1371/journal.pone.0007786)
Supplement: Table S1 — Comparison of the phenotypes of Slitrk6-knockout mice and those of neurotrophin/Ntrk knockout mice. (0.03 MB DOC) [file pone.0007786.s001.doc]

Table S1. Comparison of the phenotypes of *Slitrk6*-knockout mice and those of neurotrophin/Ntrk knockout mice.

|  | Reduction in neuronal cell number in newborn | | baso-apical gradient of neuronal loss in SG |
| --- | --- | --- | --- |
| knockout mice | SG | VG |
| *Slitrk6* | 47% | 23% | none |
| *Bdnf* | 7% | 80-85% | severer in apex |
| *Ntf3* | 85% | 23% | severer in base |
| *Ntrk2* | 15-20% | 56-85% | severer in apex |
| *Ntrk3* | 51-66% | 16-29% | severer in base |

Values are presented as % reduction compared to the neuronal number of the wild-type mouse. The data of neurotrophin and Ntrk receptor knockout mice are taken from Fritzsch et al., 2004. SG, spiral ganglon; VG, vestibular ganglion.
