# Supplementary figures and images for: Disorganized Innervation and Neuronal Loss in the Inner Ear of Slitrk6-Deficient Mice
Source: PLoS One. 2009 Nov 11;4(11):e7786. doi: 10.1371/journal.pone.0007786 (PMC2777407; doi:10.1371/journal.pone.0007786)

Fig. S1

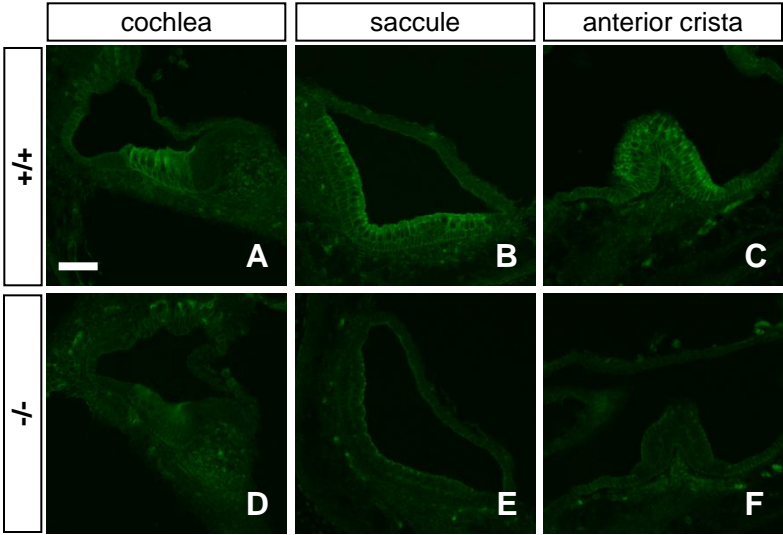

Supplement: Figure S1 — Immunohistochemistry for Slitrk6. The polyclonal antibody raised against the carboxy-terminal region of mouse Slitrk6 specifically recognizes endogenous Slitrk6 protein. Cochlea (A, D), saccule (B, E), and anterior crista (C, F) of Slitrk6 +/+(A–C) and Slitrk6 −/− (D–F) mice at P0. Positive signals on sensory epithelia disappear in Slitrk6-deficient mice. Scale bar, 50 µm. (0.69 MB PDF) [file pone.0007786.s002.pdf]

Fig. S2

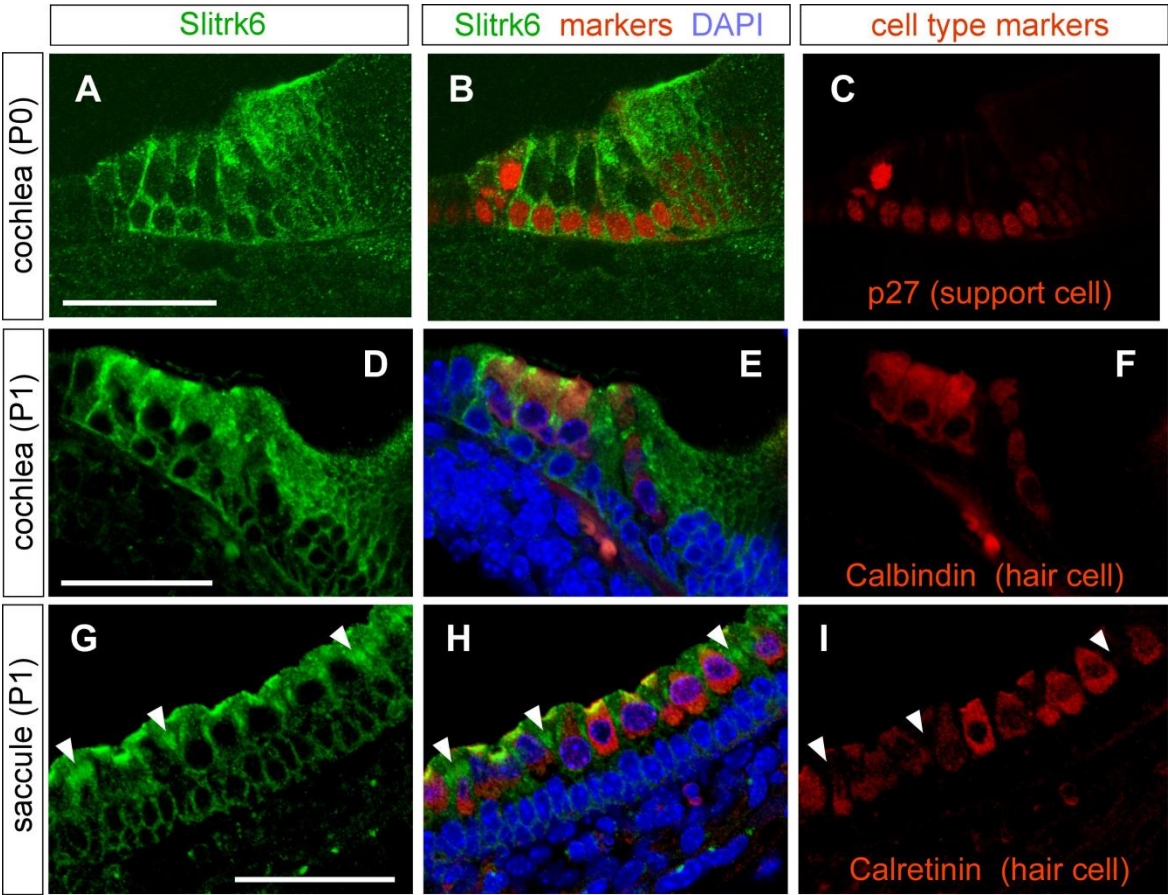

Supplement: Figure S2 — Immunostaining of newborn inner ear sensory epithelia with Slitrk6 and epithelium cell type markers. Double immunohistochemistry for Slitrk6 (green; A, D, G), and supporting cell marker p27 (red; C) or hair cell markers Calbindin (red; F) and Calretinin (red; I) on transverse sections of the P0 organ of Corti (A–C), P1 organ of Corti (D–F) and saccular macula (G–I). Immuno-staining was carried out on paraffin sections by using rabbit polyclonal anti-Slitrk6, and mouse monoclonal anti-p27 (BD Transduction Labs, San Diego, CA), anti-Calbindin (Sigma) and anti-Calretinin (Millipore) antibodies. Merged images are shown in B, E, H and DAPI-stained nuclei are seen in E, H. Arrowheads in G–I indicate the strong Slitrk6 signals outside the hair cell marker signals. Scale bar, 50 µm. (0.69 MB PDF) [file pone.0007786.s003.pdf]

Fig. S3

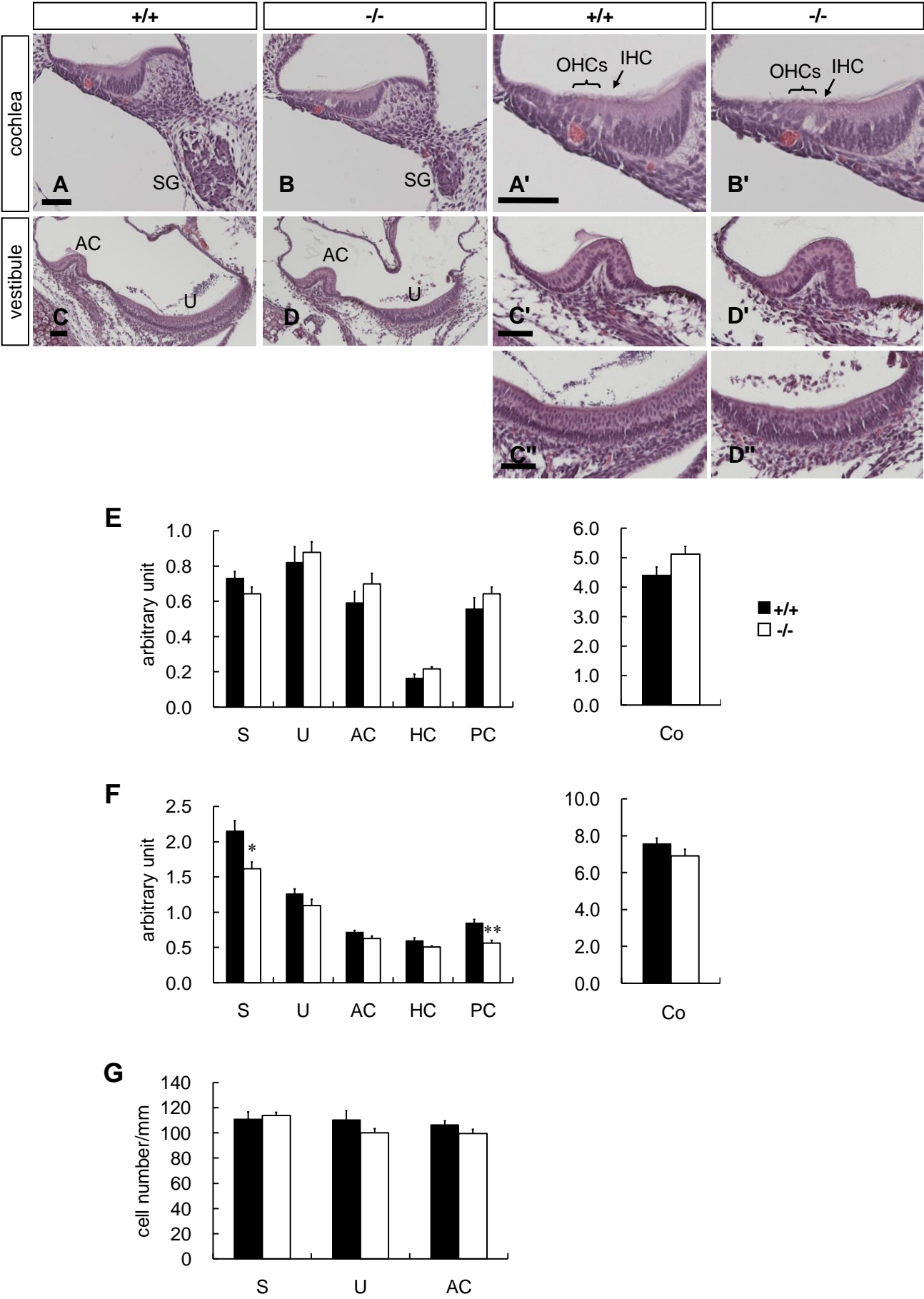

Supplement: Figure S3 — Histological examination of the inner ear sensory epithelia. H&E-stained sections of organ of Corti (A, B), and anterior crista and utricle (C, D) of Slitrk6 +/+ (A, C) and Slitrk6 −/− (B, D) mice at P0. Higher magnifications of A, B, C, and D are shown in A', B', C', C', D' and D'. The organization of the organ of Corti in the Slitrk6-deficient mice is not clearly different from that of wild-type littermates in a light microscopic analysis of H&E-stained sections (A, B). In both the wild-type and Slitrk6-deficient mice, one row of inner hair cells and three rows of outer hair cells can be identified in all turns, and the differentiation of these cells in the mutant animals appears normal (A', B'). The organization of the anterior crista and utricle also appears normal in Slitrk6-deficient mice (C, D). Higher magnification views of the anterior crista (C', D') and utricle (C', D') are not clearly different between wild-type and knockout mice. Scale bars, 50 µm. Sizes of sensory epithelia in wild-type (+/+) and Slitrk6-deficient (−/−) at E13.5 (E; +/+, N = 5; −/−, N = 5) and E16.5 (F; +/+, N = 7; −/−, N = 7). The sizes were measured in every fourth of serially prepared H&E-stained sections (thickness: 6 µm) that include the entire sensory epithelia. The sizes are indicated as the summed lumenal surface lengths of the sensory epithelia in the serial sections. A common arbitrary unit is used between (E) and (F). * p<0.05, ** p<0.01, Student's t-test. (G) Densities of the hair cells in the vestibular sensory epithelia at E16.5. The cell densities were measured by counting Myosin VIIa immuno-stained cells per unitary length (1 mm) in the serial sections. There were no significant differences in the hair cell densities between wild-type and Slitrk6-deficient mice. AC, anterior crista; Co, cochlea; HC, horizontal crista; IHC, inner hair cell; OHC, outer hair cell; PC, posterior crista; S, saccular macula; SG, spiral ganglion; U, utricle. (0.69 MB PDF) [file pone.0007786.s004.pdf]

Fig. S4

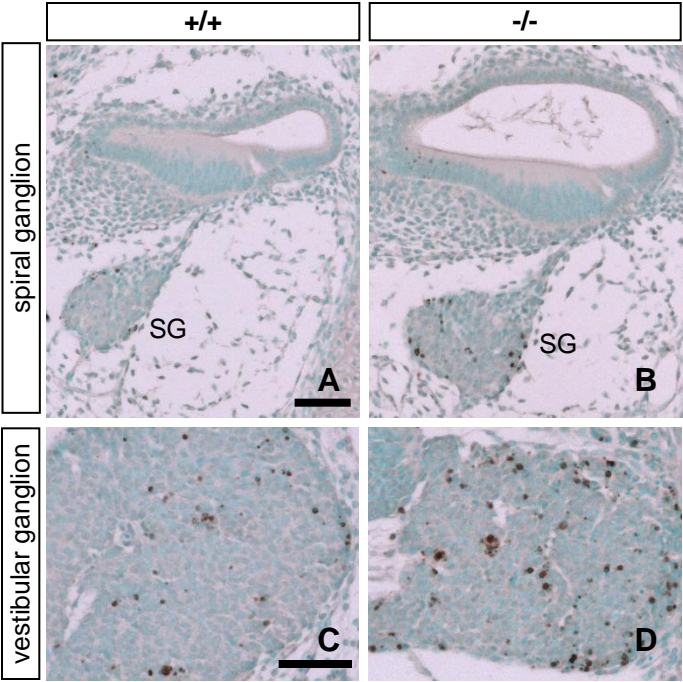

Supplement: Figure S4 — Confirmation of the cell death in the spiral and vestibular ganglia by the TUNEL method. TUNEL-stained sections of the cochlea (A, B) at E16.5, and vestibular ganglia at E13.5 (C, D) of Slitrk6 +/+ (A, C) and Slitrk6 −/− (B, D) mice. TUNEL staining was carried out using Apoptag peroxidase in situ apoptosis detection kit (Chemicon, Temecula, CA) in accordance with the manufacturer's instructions. The positive signals were visualized by a peroxidase-diaminobenzidine reaction, and then the sections were counterstained with methyl green. TUNEL-positive signals are increased in Slitrk6-deficient mice in both the spiral and vestibular ganglia. SG, spiral ganglion. Scale bars, 50 µm. (0.69 MB PDF) [file pone.0007786.s005.pdf]

Fig. S5

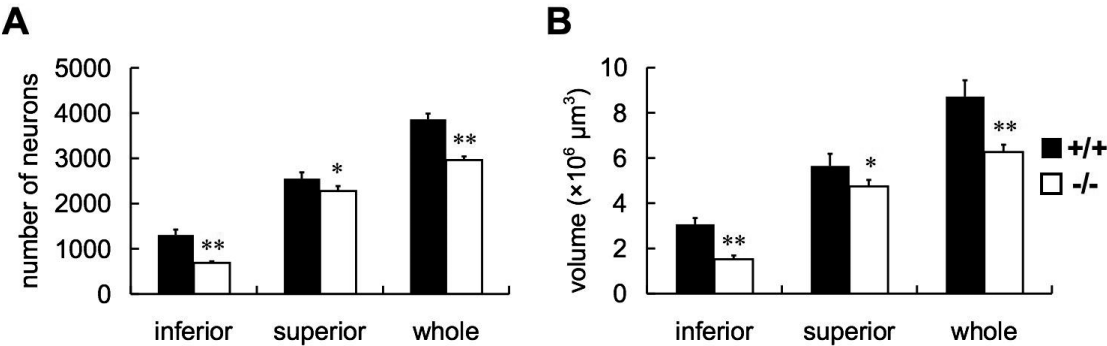

Supplement: Figure S5 — Reduction in both the number of neurons and volume of vestibular ganglion in Slitrk6-deficient mice was more pronounced in inferior vestibular ganglion than superior vestibular ganglion. Number of neurons (A) and volume (B) of the vestibular ganglion at P0. Values are presented as Mean + SD of 4 wild-type (black bar) and 3 knockout (white bar) mice. *, p<0.05; **, p<0.01 in Student's t-test. (0.69 MB PDF) [file pone.0007786.s006.pdf]

Fig. S6

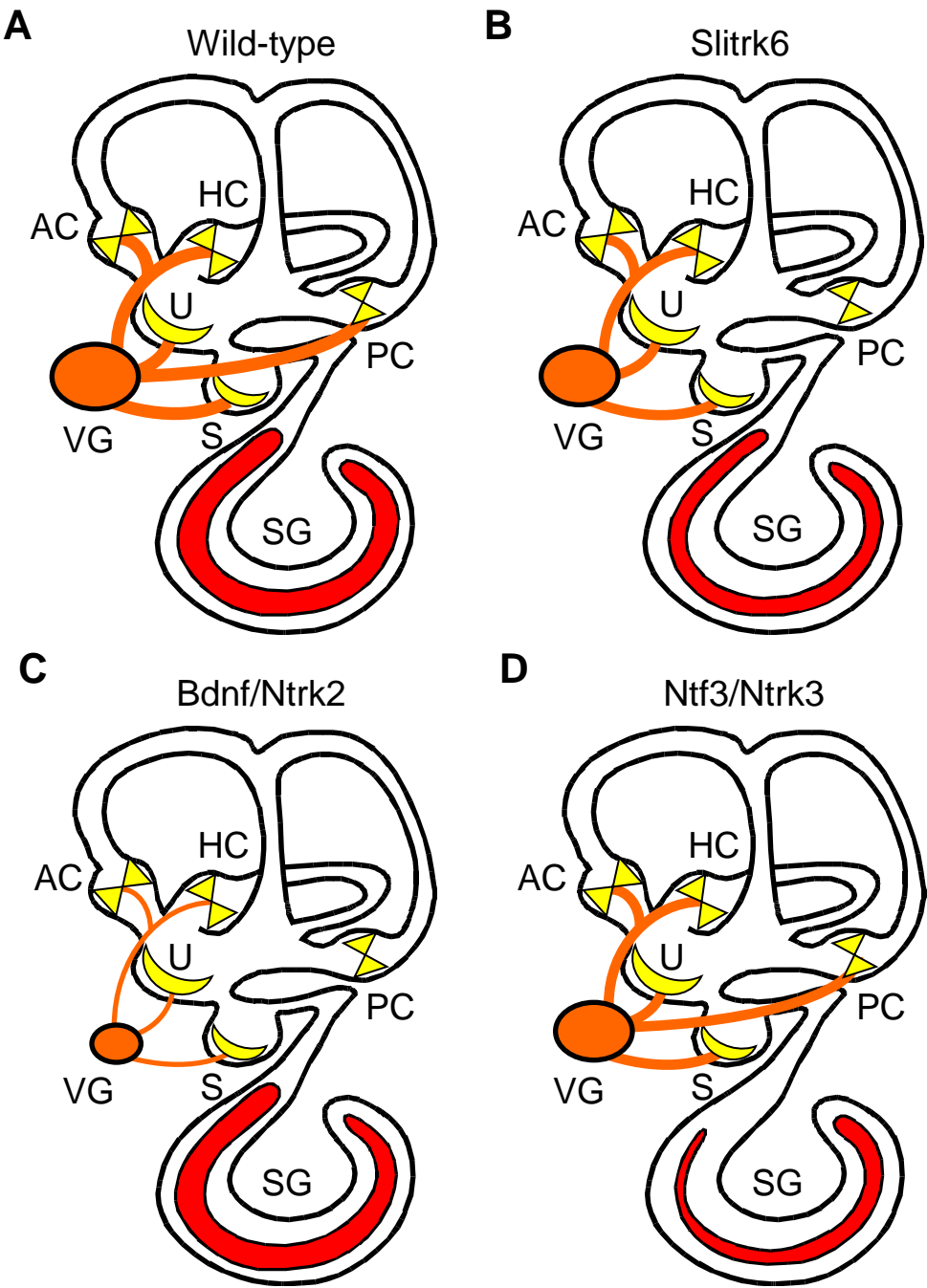

Supplement: Figure S6 — Comparisons of the inner ear phenotypes of Slitrk6-knockout mice and those of neurotrophin/Ntrk-knockout mice. Schematic drawing of the inner ear phenotypes of wild-type (A), Slitrk6-knockout (B), Bdnf/Ntrk2-knockout (C) and Ntf3/Ntrk3-knockout mice (D). Cristae and maculae are indicated in yellow. Vestibular ganglia and their projections are shown in orange and spiral ganglia are shown in red. The number of vestibular ganglion neurons is severely decreased in Bdnf- and Ntrk2-null mutants (C), whereas a reduction in the number of the spiral ganglion neurons (indicated by thick red lines in cochlea) is prominent in Ntf3- and Ntrk3-null mutants (D) [21], [26], [27]. In the cochlea, Ntf3- and Ntrk3-null mutants predominantly lose spiral ganglion neurons in the basal turn (D), and neuronal loss in Bdnf- and Ntrk2-null mutants is most obvious in the apex (C) [16], [21], [28], [29]. In the Slitrk6-knockout mice, neuronal loss was more pronounced in the cochlea than in the vestibule, but Slitrk6-deficient cochleae showed no phenotypic gradient in neuronal loss along the baso-apical axis (B). AC, anterior crista; HC, horizontal crista; PC, posterior crista; S, saccule; SG, spiral ganglion; U, utricle; VG, vestibular ganglion. (0.69 MB PDF) [file pone.0007786.s007.pdf]

Fig. S7

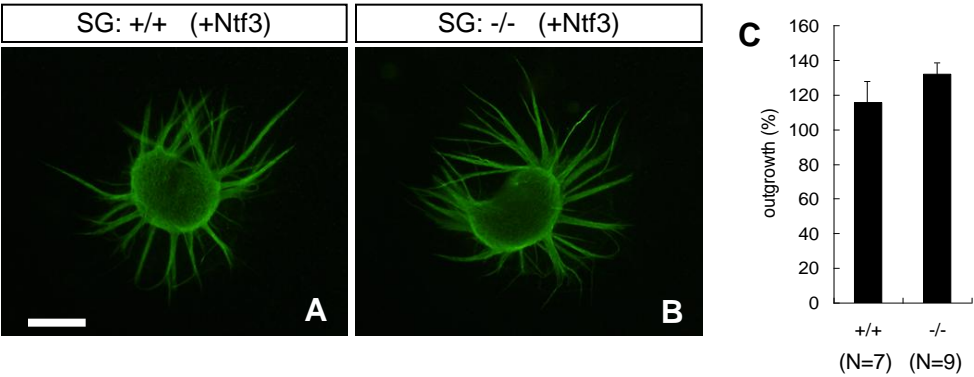

Supplement: Figure S7 — Spiral ganglion neurons of Slitrk6-deficient mice can extend neurites identical to those of wild-type mice in the presence of neurotrophin. Spiral ganglia from E14.5 wild-type (+/+) and Slitrk6-knockout (−/−) mice were embedded in the collagen gel (Nitta Gelatin) and cultured in neurobasal medium (Invitrogen) containing 2 mM L-glutamine, B27 supplement (Invitrogen) and 20 ng/ml of Neurotrophin-3 (+Ntf3, PeproTech EC, London, UK) for 48 hours. Neurites were visualized by neurofilament immunostaining (Sigma, green). Spiral ganglion neurons of Slitrk6-knockout mice (B) can extend their neurites as those of wild-type mice (A). Scale bar, 200 µm. (C) Measurement of neurite outgrowth (%) of spiral ganglia. The graphs represent mean + SEM. Neurite outgrowth of spiral ganglia was not significantly different between wild-type and Slitrk6-deficient mice. (0.69 MB PDF) [file pone.0007786.s008.pdf]

Fig. S8

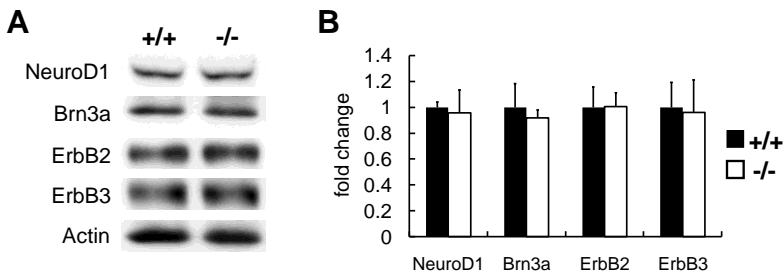

Supplement: Figure S8 — Amounts of proteins that are known to mediate inner ear sensory neural development in Slitrk6-knockout mice. (A) Western blot analysis of proteins extracted from E14.5 cochlea (includes cochlear sensory epithelium and spiral ganglion). Goat polyclonal anti-NeuroD1 (Santa Cruz Biotechnology), mouse monoclonal anti-Brn3a (Santa Cruz Biotechnology) and anti-ErbB3 (Thermo Fisher Scientific, Fremont, CA), and rabbit polyclonal anti-ErbB2 (Thermo Fisher Scientific) and anti-β-actin (Sigma) antibodies were used as primary antibodies. (B) The graphs represent the mean + SD of 3 independent analyses. There were no significant differences in the expression of NeuroD1, Brn3a, ErbB2, or ErbB3 between wild-type (+/+) and Slitrk6 knockout (−/−) mice. (0.69 MB PDF) [file pone.0007786.s009.pdf]
